# Predictors of Self‐Care Behaviours Among Patients With Chronic Disease: A Multicountry Survey

**DOI:** 10.1155/nrp/5262222

**Published:** 2026-05-19

**Authors:** Hanan Abdelrahman, Mohammad Al Qadire, Hamada Zehry, Suha Ballout, Hala Aljezawi, Laura L. Hayman

**Affiliations:** ^1^ College of Nursing and Health Sciences, University of Massachusetts Boston, Boston, Massachusetts, USA, umb.edu; ^2^ Faculty of Nursing, Suez Canal University, Ismailia, Egypt, scuegypt.edu.eg; ^3^ Princess Salma Faculty of Nursing, Al Al-Bayt University, Mafraq, Jordan, aabu.edu.jo; ^4^ Faculty of Medicine, Horus University-Egypt, Damietta, Egypt; ^5^ Al Mafraq Governmental Hospital, Ministry of Health, Mafraq, Jordan, moh.gov.jo

**Keywords:** adult, chronic diseases, disease management, Middle East, self-care, self-efficacy

## Abstract

**Aim:**

This study aims to assess self‐care behaviours and their predictors among patients with chronic diseases in three Middle Eastern countries.

**Design:**

A cross‐sectional study was conducted.

**Methods:**

The study was conducted in hospitals across Oman, Jordan and Egypt. A convenience sample of 1598 adults with chronic diseases was recruited. Data were collected using a structured questionnaire, including sociodemographic characteristics, the Arabic version of the Self‐Care of Chronic Illness Inventory and the Self‐Care Self‐Efficacy Scale. Multiple regression models identified significant predictors of self‐care behaviours.

**Results:**

The sample had a mean age of 48.1 years (SD = 15.8), with 56.2% male. Diabetes mellitus (23.7%) was the most common condition, followed by cerebrovascular accidents (16.9%) and cardiovascular diseases (16.3%). Self‐care behaviours were suboptimal, with mean scores below 70% across all subscales. Self‐efficacy was the strongest predictor, followed by educational level, gender and regular healthcare visits. Participants from Jordan and Oman reported higher self‐care engagement than those from Egypt.

**Conclusions:**

Findings highlight the need for targeted interventions to enhance self‐efficacy, improve health literacy and address healthcare disparities. Culturally tailored education programmes and structured self‐management support are essential to promote better self‐care practices among patients with chronic diseases.

## 1. Introduction

Chronic diseases, including cardiovascular diseases, diabetes and chronic respiratory conditions, are among the leading causes of mortality and morbidity worldwide, accounting for 71% of the global deaths [1]. Effective management of these diseases requires continuous patient engagement in self‐care behaviours, including medication adherence, lifestyle modifications, symptom monitoring and timely medical consultations [2]. These behaviours are essential for improving patient outcomes, enhancing quality of life and reducing healthcare utilisation [3]. Research has demonstrated that structured self‐management support programmes can significantly reduce hospitalisation, emergency department visits and healthcare costs, reinforcing the importance of self‐care in chronic disease management [3–5]. Despite its well‐documented benefits, adherence to self‐care remains a major challenge, particularly in low‐ and middle‐income countries, where barriers such as limited healthcare access, financial constraints, and educational gaps hinder effective disease management [6, 7].

The management of chronic diseases in these settings is further complicated by variations in national guidelines, inconsistencies in monitoring frameworks, and disparities in healthcare system capacities compared with high‐income countries [6, 8]​. These systemic differences can lead to significant inequalities in access to medications, healthcare services and self‐care education, making it difficult for patients to engage in consistent disease‐management practices [9, 10]. Several demographic and clinical factors influence self‐care behaviours, including age, sex, education level, socioeconomic status, cognitive function, psychological well‐being, and social support systems [11, 12]. Older adults often demonstrate better self‐care management because of their experiences with disease progression and symptom recognition. However, they may struggle with self‐care maintenance and monitoring due to cognitive decline, physical limitations and reliance on carers [9]. Gender differences also play a role, with studies indicating that women are more likely to adhere to preventive behaviours, whereas men tend to be more confident in managing acute symptoms [8, 13]. Education level is a significant determinant of self‐care engagement, as it is closely linked to health literacy. Patients with higher educational levels tend to have better health literacy, enabling them to understand their conditions, follow treatment recommendations and adopt appropriate self‐care behaviours [3]. Conversely, lower educational levels are associated with limited health literacy, which can hinder a patient’s ability to interpret medical instructions, recognise symptoms and seek timely medical attention [9]. This disparity suggests that targeted interventions are necessary to improve health literacy, particularly among populations with lower educational attainment.

Socioeconomic status also plays a crucial role in self‐care behaviours, as financial constraints can limit access to essential healthcare resources, medications and nutritious foods, all of which are vital for effective disease management [3]. Patients from lower‐income backgrounds may experience additional challenges in maintaining regular follow‐ups and adhering to prescribed treatments, owing to economic hardships [14]. Psychosocial factors, such as depression, anxiety and self‐efficacy, also influence adherence to self‐care. Depression has been linked to reduced medication adherence, low engagement in physical activity, and poor dietary habits, all of which can negatively affect chronic disease management [14]. Self‐efficacy, or a patient’s confidence in their ability to manage their condition, has been identified as a key mediator between knowledge and self‐care behaviours, further emphasising the importance of psychological interventions along with traditional educational approaches [15]. Social support systems, including family and carers, are also essential for reinforcing self‐care behaviours. Patients who received strong family support tended to have better adherence to medication, symptom monitoring, and disease management than those who did not [13]. In many cases, carer involvement plays a crucial role in facilitating disease management, particularly in older adults and in individuals with multiple chronic conditions [9, 16].

Importantly, self‐care is increasingly recognised as a multidimensional and relational process rather than a solely individual responsibility. Emerging evidence demonstrates that self‐care behaviours are shaped by dynamic interactions between patients, carers and healthcare professionals, rather than individual competence alone [16]. The quality of patient–provider communication has been shown to directly influence adherence, decision‐making, and long‐term disease management outcomes [16]. Similarly, carer involvement plays a critical role in supporting daily self‐care activities, particularly among patients with complex or multiple chronic conditions [17]. Health literacy is another key determinant, as limited understanding of disease processes and treatment recommendations can significantly hinder effective self‐care engagement [17]. In addition, psychological burden—including anxiety, depression and illness‐related distress—has been consistently associated with reduced motivation and poorer adherence to self‐care behaviours. Recent studies, particularly in chronic gastrointestinal conditions, further emphasise that self‐care is co‐constructed within social and clinical contexts, where relational support and healthcare system interactions shape patients’ capacity to manage their illness effectively [16–18]. This broader perspective highlights the need to move beyond individual‐level models and incorporate relational and contextual determinants when examining self‐care behaviours.

Despite growing evidence on self‐care behaviours in chronic illness, most studies have focused on single‐disease populations and have been conducted predominantly in Western or high‐income settings. This limits the generalisability of findings to patients with multiple chronic conditions and to regions with different healthcare infrastructures and sociocultural contexts. In addition, few studies have examined self‐care behaviours across multiple countries within the Middle East, where variations in healthcare systems, economic resources, and cultural norms may significantly influence patient engagement in self‐care. A multicounty approach allows for the identification of both shared and context‐specific predictors, providing a more comprehensive understanding of self‐care behaviours beyond disease‐specific models. Therefore, this study was designed to examine self‐care behaviours and their predictors across diverse chronic conditions in three Middle Eastern countries, with the hypothesis that demographic, clinical and psychosocial factors—including self‐efficacy—would significantly predict self‐care behaviours across settings.

## 2. Methods

### 2.1. Design

This study employed a descriptive cross‐sectional design.

### 2.2. Settings

The study was conducted in three hospitals across Arab countries: Sultan Qaboos University Hospital in Muscat, Oman; Al Basheer Hospitals in Amman, Jordan; and Damietta General Hospital and Damietta Specialized Hospital in Damietta, Egypt. As tertiary care centres, these hospitals provide specialised medical services for a wide range of chronic conditions. They serve both inpatient and outpatient populations and offer essential medical and nursing care within their healthcare systems. The sample distribution across participating centres was as follows: Sultan Qaboos University Hospital (Oman) (*n* = 350), Al Basheer Hospitals (Jordan) (*n* = 620), Damietta General Hospital (Egypt) (*n* = 324), and Damietta Specialized Hospital (Egypt) (*n* = 304).

### 2.3. Sample and Sampling

The study included patients diagnosed with chronic illnesses who were receiving either treatment or follow‐up care at designated hospitals. The participants were recruited using convenience sampling. Eligibility criteria required individuals to be at least 18 years old, able to read and comprehend Arabic, and have been managing a chronic condition for at least 1 year. Voluntary consent was required to participate in the study. A broad spectrum of chronic diseases qualified for inclusion, such as chronic obstructive pulmonary disease (COPD), diabetes mellitus, heart failure, hypertension, asthma, osteoporosis, kidney failure and various forms of cancer. However, individuals facing specific barriers to participation were excluded. This included those with severe illnesses that could compromise their ability to engage in the study, cognitive impairments preventing them from understanding the study’s objectives or completing the questionnaire independently, and significant communication challenges, including deafness or blindness.

### 2.4. Sample Size Calculation

The sample size was calculated a priori using G∗Power 3.1 for linear multiple regression (fixed model, *R* ^2^ deviation from zero), with the primary outcome being self‐care behaviour. Assuming a small‐to‐medium effect size of *f* ^2^ = 0.10, a significance level of *α* = 0.05, statistical power of 0.95, and up to 15 predictor variables in the model, the minimum required sample size was 225 participants. To account for potential nonresponse and missing data (approximately 20%) and to allow for comparisons across the three participating countries, we aimed to recruit at least 270 participants per country (total ≈ 810). The final achieved sample of 1598 participants, therefore, exceeded the minimum required sample size and provided adequate power for the planned analyses.

### 2.5. Instruments

The data‐collection instrument was structured into three main sections. The first section focused on gathering sociodemographic information such as participants′ age, sex, marital status, educational background, employment status, income level, medical diagnosis and duration of illness.

The second section included the Self‐Care of Chronic Illness Inventory—Arabic version (SC‐CII‐Ar) [19], which is a validated tool designed to assess self‐care behaviours among individuals with chronic conditions. Adapted from the original Self‐Care of Chronic Illness Inventory (SC‐CII) [20]. The SC‐CII‐Ar comprises three subscales: Self‐Care Maintenance (eight items), Self‐Care Monitoring (five items), and Self‐Care Management (seven items), each evaluating different aspects of self‐care behaviours. Participants responded using a 5‐point Likert scale, with higher scores reflecting better self‐care practices. The total score for this section was calculated by summing the responses across all the 20 items. To facilitate interpretation, raw scores were transformed to a 0–100 scale, where higher scores represent better self‐care abilities. The SC‐CII‐Ar demonstrated strong psychometric properties, including high internal consistency (Cronbach’s alpha > 0.80) and excellent test–retest reliability (ICC > 0.90) [19].

The third section of the questionnaire incorporated the Self‐Care Self‐Efficacy Scale (SCSES) [21], a standardised tool used to measure patients′ confidence in their ability to engage in self‐care activities. It assesses self‐efficacy in three key areas: self‐care maintenance (routine health behaviours), self‐care monitoring (recognising symptoms and changes in health status), and self‐care management (responding to symptom exacerbations). The scale consists of 10 items, each rated on a 5‐point Likert scale, with higher scores indicating greater self‐efficacy. The SCSES has been validated across multiple cultural contexts, demonstrating strong internal reliability (Cronbach’s alpha > 0.90) and measurement equivalence across diverse patient populations [2].

The scale was translated into Arabic using a forward–backward translation process in accordance with established guidelines, including independent forward translation, synthesis, back translation, expert committee review, and pilot testing to ensure linguistic and cultural appropriateness [19]. The psychometric evaluation of the Arabic version demonstrated strong validity and reliability, with confirmatory factor analysis supporting the scale structure. Internal consistency was high, with Cronbach’s *α* of 0.89 for self‐care maintenance, 0.82 for self‐care monitoring, and 0.86 for self‐care management, while subscale values ranged from 0.84 to 0.88 [19].

### 2.6. Data Collection

Data collection took place from 15 November 2023 to 10 July 2024. Data for this study were collected through structured, self‐administered questionnaires distributed to participants in clinical settings across three selected Middle Eastern countries. Participants were recruited using a convenience sampling approach across the participating hospitals. Eligible patients were identified on‐site in outpatient clinics and inpatient units by trained research assistants in coordination with clinical staff. Potential participants were approached during their clinical visits or hospital stay, provided with information about the study, and invited to participate. Those who agreed were enrolled after providing informed consent. No additional selection filters were applied beyond the predefined eligibility criteria. Each participant received an information sheet outlining the study objectives, procedures, potential risks and benefits, and rights as research subjects. Adequate time was given to the participants to review the information before deciding whether to participate.

The trained research assistants were available on‐site to address any questions, clarify doubts, and ensure that the participants fully understood the questionnaire items. This was particularly important for individuals with lower health literacy levels, ensuring that their responses accurately reflected their self‐care behaviours and experiences. The participants were asked to complete the questionnaire in a quiet and private setting. The estimated completion time was 20–30 min. Upon completion, participants returned the questionnaire to the research assistant.

To facilitate broad participation, data collection was scheduled at different times of the day to accommodate patients with varying availabilities.

### 2.7. Data Analysis

Data were entered and analysed using IBM SPSS Statistics for Windows, Version 30.0 (IBM Corp., Armonk, NY, USA). Missing data were assessed prior to analysis. The overall proportion of missing data was low (< 5%) across all study variables. This was partly due to the data collection procedure, whereby trained research assistants reviewed completed questionnaires on‐site to ensure completeness, thereby minimising missing responses. Assuming that the remaining missing data were missing at random (MAR), a complete‐case (listwise deletion) approach was applied in the regression analyses.

The analysis involved both descriptive and inferential statistical methods to examine self‐care behaviours and their predictors in patients with chronic disease. Descriptive statistics, including means, standard deviations, frequencies and percentages, were used to summarise demographic and clinical characteristics as well as self‐care behaviour scores across the maintenance, monitoring and management subscales. The multiple regression models were conducted to identify significant predictors of self‐care behaviours. All relevant variables, selected based on theoretical significance and existing literature, were incorporated into the constructed models to ensure a comprehensive analysis. This approach ensured a comprehensive model by accounting for potential confounders that may not have shown significant bivariate associations but could still influence the multivariable analysis. Incorporating all pertinent variables enhanced the model’s accuracy in representing predictor‐outcome relationships while minimising omitted variable bias. This methodology aligns with the recommendations of Harrell (2001) and Greenland (1989), who advocate for theory‐driven variable selection over reliance on bivariate screening to prevent bias and omission of important factors [22, 23]. Statistical significance was set at *P* < 0.05.

### 2.8. Ethical Considerations

Prior to data collection, the study was approved by relevant ethics committees within the selected settings. This study adhered to the principles of the Declaration of Helsinki. Approval was obtained from the Ethics Committee of the College of Nursing at Sultan Qaboos University (Ref. No. CON/NF/2023/9). Additionally, approvals were secured from the ethics committees of Sultan Qaboos University Hospital (REF. NO. SQU‐EC/150/2023), and the Jordan Ministry of Health (MOH/REC/2023/242).

Adequate information about the study was provided to the participants, ensuring that they fully understood its purpose, procedures and rights. Written informed consent was obtained from all participants prior to their involvement. Participation was entirely voluntary, allowing individuals to withdraw at any time without any consequences to their medical care or relationships with healthcare providers. To maintain confidentiality, only aggregate data were included in the published findings, ensuring that personal information remained protected. To reinforce data security, all collected information was stored in locked cabinets and password‐protected computers with access strictly limited to the research team.

## 3. Results

### 3.1. Participants’ Sociodemographic Characteristics

This study included 1598 participants from Egypt (39.3%, *n* = 628), Jordan (38.8%, *n* = 620) and Oman (21.9%, *n* = 350). The majority were female (56.2%, *n* = 898) with a mean age of 48.1 years (SD = 15.8) and 64.0% (n = 1022) of the participants were married. In terms of education, 50.8% (*n* = 812) had completed secondary school or lower, and 49.2% (*n* = 786) had a diploma or higher. Employment status was nearly evenly distributed, with 51.0% (*n* = 815) working and 49.0% (*n* = 783) not working. Participants reported a range of medical conditions, with diabetes mellitus (23.7%) being the most common, followed by cerebrovascular accidents (16.9%), cardiovascular diseases (16.3%) and respiratory conditions (14.2%). Additionally, 48.6% (*n* = 778) of participants reported having at least one additional chronic disease. The mean duration since the diagnosis was 10.5 years (SD = 11.7). Among the participants, 82.7% (*n* = 1322) reported having a family carer. Regular healthcare visits were reported by 79.1% (*n* = 1264). Healthcare providers were the primary source of disease‐related information for 81.5% (*n* = 1303) of participants. The mean monthly income was 684 (SD = 1548) U.S. dollars. Table 1 provides further details on the participants’ characteristics.

**TABLE 1:** Participants’ sociodemographic characteristics (*n* = 1598).

Characteristic	Frequency (%)	Mean (SD)
Country		
Egypt	628 (39.3)	
Jordan	620 (38.8)	
Oman	350 (21.9)	
Gender		
Male	700 (43.8)	
Female	898 (56.2)	
Marital status		
Married	1022 (64.0)	
Not married	576 (36.0)	
Educational level		
Secondary school or lower	812 (50.8)	
Diploma or higher	786 (49.2)	
Work		
Working	783 (49.0)	
Not working	815 (51.0)	
Category of diagnosis		
Haematology	137 (8.6)	
Cancers	70 (4.4)	
Diabetes mellitus	378 (23.7)	
Cardiovascular	260 (16.3)	
Renal	111 (6.9)	
Cerebrovascular accident	270 (16.9)	
Respiratory	227 (14.2)	
Gastrointestinal	145 (9.1)	
Family carer		
Yes	1322 (82.7)	
No	276 (17.3)	
Having additional chronic diseases		
Yes	778 (48.6)	
No	820 (51.4)	
Healthcare visit schedule		
Regular	1264 (79.1)	
Rarely	334 (20.9)	
Source of disease information		
Healthcare provider	1303 (81.5)	
Family and friends	136 (8.5)	
Social media	159 (10.0)	
Age (years)		48.1 (15.8)
Diagnosis duration (years)		10.5 (11.7)
Monthly income ($)		684 (1548)

### 3.2. Self‐Care Behaviours

Patients reported relatively low mean scores (< 70%) on the subscales of the Self‐Care Inventory. The mean scores for Self‐Care Maintenance were 60.8 (SD = 16.9), 64.6 (SD = 19.8) for Self‐Care Monitoring, and 66.8 (SD = 19.9) for Self‐Care Management. However, the mean score on the self‐efficacy scale was lowest at 59.1 (SD = 21.7).

## 4. Predictors of Self‐Care Behaviours

### 4.1. Self‐Care Maintenance

Regression analysis identified several significant predictors of self‐care maintenance among patients with chronic diseases, explaining 39% of the variance in self‐care maintenance scores (*R* ^2^ = 0.39; adjusted *R* ^2^ = 0.38). For self‐care maintenance, statistically significant predictors included gender, educational level, regular healthcare visits, specific disease categories, source of information, country and self‐efficacy (Table 2).

**TABLE 2:** Predictors of self‐care maintenance among patients with chronic diseases (*n* = 1598).

Predictor	B	Std. error	Beta	*t*	*p*	95% confidence interval	
(Constant)	34.135	2.127		16.046	**< 0.001**	29.962	38.308
Gender (male with reference to female)	−1.572	0.694	−0.046	−2.265	**0.024**	−2.934	−0.211
Age	−0.014	0.025	−0.013	−0.534	0.593	−0.063	0.036
Educational level (diploma or higher with reference to secondary school or lower)	2.989	0.783	0.089	3.817	**< 0.001**	1.453	4.525
Healthcare visit schedule (regular with reference to not rarely)	6.208	0.929	0.150	6.681	**< 0.001**	4.385	8.030
Category of diagnosis (reference category: haematology)							
Cancer	−3.480	2.082	−0.041	−1.671	0.095	−7.565	0.604
Diabetes mellitus	−2.862	1.419	−0.072	−2.017	**0.044**	−5.646	−0.079
Cardiovascular	−3.721	1.572	−0.082	−2.367	**0.018**	−6.804	−0.637
Renal	0.901	1.896	0.014	0.475	0.635	−2.819	4.621
Cerebrovascular accident	−4.639	1.558	−0.104	−2.977	**0.003**	−7.695	−1.583
Respiratory	−2.731	1.558	−0.057	−1.753	0.080	−5.787	0.325
Gastrointestinal	−0.722	1.678	−0.012	−0.430	0.667	−4.013	2.569
Source of disease information (reference category: healthcare provider)							
Family and friends	−5.172	1.276	−0.085	−4.053	**< 0.001**	−7.675	−2.669
Social media	−5.655	1.176	−0.100	−4.810	**< 0.001**	−7.961	−3.349
Country (reference category: Egypt)							
Jordan	3.233	0.878	0.094	3.685	**< 0.001**	1.512	4.955
Oman	6.960	1.079	0.166	6.453	**< 0.001**	4.845	9.076
Self‐care confidence	0.379	0.018	0.487	20.995	**< 0.001**	0.344	0.415

*Note:* *R* ^2^ = 0.39, adjusted *R* ^2^ = 0.38. Bold values are significant at *p* < 0.05.

Gender was a significant predictor, with males reporting lower self‐care maintenance scores than females (*B* = −1.572, *p* = 0.024). A higher educational level (diploma or higher) was associated with better self‐care maintenance (*B* = 2.989, *p* < 0.001). Regular healthcare visits were also a strong predictor of improved self‐care maintenance (*B* = 6.208, *p* < 0.001). Patients with cerebrovascular disease (*B* = −4.639, *p* = 0.003), cardiovascular disease (*B* = −3.721, *p* = 0.018), and diabetes mellitus (*B* = −2.862, *p* = 0.044) exhibited significantly lower self‐care maintenance scores than those with haematological conditions. However, no significant differences were observed in patients with cancer or renal, respiratory, or gastrointestinal disease. The source of disease information also played a role, as patients relying on family and friends (*B* = −5.172, *p* < 0.001) and social media (*B* = −5.655, *p* < 0.001) had significantly lower self‐care maintenance scores than those receiving information from healthcare providers. Geographic differences were also evident, with patients in Jordan (*B* = 3.233, *p* < 0.001) and Oman (*B* = 6.960, *p* < 0.001) reporting higher self‐care maintenance than those in Egypt. Finally, self‐efficacy emerged as the strongest predictor of self‐care maintenance (*B* = 0.379, *p* < 0.001), indicating that higher confidence in managing one’s health was associated with better self‐care behaviours.

### 4.2. Self‐Care Monitoring

Regression analysis identified several significant predictors of self‐care monitoring among patients with chronic diseases, explaining 40% of the variance in self‐care monitoring scores (adjusted *R* ^2^ = 0.40). For self‐care monitoring, statistically significant predictors included gender, educational level, regular healthcare visits, specific disease categories, source of information, country and self‐efficacy (Table 3).

**TABLE 3:** Predictors of self‐care monitoring among patients with chronic diseases (*n* = 1598).

Predictor	B	Std. error	Beta	*t*	*p*	95% confidence interval	
(Constant)	30.772	2.745		11.212	**< 0.001**	25.388	36.156
Gender (male with reference to female)	−3.416	0.839	−0.086	−4.074	**< 0.001**	−5.061	−1.771
Age	0.021	0.031	0.017	0.688	0.492	−0.040	0.083
Educational level (diploma or higher with reference to secondary school or lower)	4.060	0.958	0.103	4.238	**< 0.001**	2.181	5.940
Marital status (married with reference to not married)	1.648	0.865	0.040	1.905	0.057	−0.049	3.344
Healthcare visit schedule (regular with reference to not rarely)	6.906	1.119	0.143	6.173	**< 0.001**	4.712	9.101
Category of diagnosis (reference category: haematology)							
Cancer	−0.665	2.567	−0.006	−0.259	0.795	−5.700	4.369
Diabetes mellitus	−4.943	1.698	−0.107	−2.912	**0.004**	−8.273	−1.613
Cardiovascular	−4.706	1.878	−0.089	−2.506	**0.012**	−8.390	−1.023
Renal	0.087	2.235	0.001	0.039	0.969	−4.297	4.470
Cerebrovascular accident	−7.200	1.847	−0.140	−3.897	**< 0.001**	−10.824	−3.576
Respiratory	−2.731	1.857	−0.049	−1.470	0.142	−6.374	0.913
Gastrointestinal	−1.504	2.001	−0.022	−0.752	0.452	−5.428	2.420
Source of disease information (reference category: healthcare provider)							
Family and friends	−4.966	1.526	−0.070	−3.254	**0.001**	−7.960	−1.972
Social media	−5.843	1.382	−0.089	−4.228	**< 0.001**	−8.553	−3.132
Country (reference category: Egypt)							
Jordan	7.540	1.068	0.189	7.059	**< 0.001**	5.445	9.635
Oman	8.568	1.381	0.167	6.204	**< 0.001**	5.859	11.276
Work (with reference to not working)	−0.761	0.960	−0.019	−0.792	0.428	−2.644	1.123
Having family carer (yes with reference to no)	−1.021	1.106	−0.020	−0.922	0.356	−3.191	1.150
Self‐efficacy score	0.457	0.021	0.505	21.424	**< 0.001**	0.416	0.499
Monthly income	0.120	0.010	−0.020	−0.899	0.369	−0.021	0.001

*Note:* *R* ^2^ = 0.41, adjusted *R* ^2^ = 0.40. Bold values are significant at *p* < 0.05.

Gender was a significant factor, with males demonstrating lower self‐care monitoring than females (*B* = −3.416, *p* < 0.001). Educational attainment was also significant, as individuals with a diploma or higher reported significantly better self‐care monitoring than those with lower educational levels (*B* = 4.060, *p* < 0.001). Regular healthcare visits emerged as a strong predictor, with those attending healthcare appointments consistently exhibiting higher self‐care monitoring (*B* = 6.906, *p* < 0.001). Several disease categories were associated with variations in self‐care monitoring. Patients with cerebrovascular disease (*B* = −7.200, *p* < 0.001), cardiovascular disease (*B* = −4.706, *p* = 0.012) and diabetes mellitus (*B* = −4.943, *p* = 0.004) reported significantly lower self‐care monitoring scores than those with haematological conditions. However, no significant differences were observed among the patients with cancer, renal disease, respiratory conditions, or gastrointestinal disorders. The source of the health information is also a significant determinant. Patients who primarily relied on family and friends (*B* = −4.966, *p* = 0.001) or social media (*B* = −5.843, *p* < 0.001) exhibited significantly lower self‐care monitoring scores than those who received information from healthcare providers. Furthermore, geographic differences were evident, with patients in Jordan (*B* = 7.540, *p* < 0.001) and Oman (*B* = 8.568, *p* < 0.001) demonstrating significantly higher self‐care monitoring compared to those in Egypt. Among all predictors, self‐efficacy emerged as the strongest factor influencing self‐care monitoring (*B* = 0.457, *p* < 0.001), indicating that greater confidence in self‐care was associated with enhanced monitoring behaviours.

### 4.3. Self‐Care Management

Regression analysis identified significant predictors of self‐care management among patients with chronic disease, explaining 35% of the variance in self‐care management scores (adjusted *R* ^2^ = 0.35). For self‐care management, statistically significant predictors included gender, age, healthcare visit patterns, specific disease categories, source of information, country and self‐efficacy (Table 4).

**TABLE 4:** Predictors of self‐care management among patients with chronic diseases (*n* = 1598).

Predictor	B	Std. error	Beta	*t*	*p*	95% confidence interval	
(Constant)	30.582	2.603		11.747	< 0.001	25.475	35.688
Gender (male with reference to female)	−1.805	0.851	−0.045	−2.120	**< 0.001**	−3.474	−0.135
Age	0.103	0.031	0.082	3.319	**< 0.001**	0.042	0.164
Educational level (diploma or higher with reference to secondary school or lower)	4.349	0.962	0.109	4.521	0.057	−0.068	4.398
Healthcare visit schedule (regular with reference to not rarely)	2.165	1.138	0.044	1.902	**< 0.001**	2.042	6.235
Category of diagnosis (reference category: haematology)							
Cancer	−3.005	2.537	−0.030	−1.184	0.236	−7.980	1.971
Diabetes mellitus	−4.851	1.735	−0.104	−2.796	**0.005**	−8.254	−1.449
Cardiovascular	−0.724	1.921	−0.013	−0.377	0.706	−4.492	3.045
Renal	6.387	2.322	0.082	2.750	**0.006**	1.832	10.942
Cerebrovascular accident	−0.669	1.907	−0.013	−0.351	0.726	−4.409	3.071
Respiratory	0.503	1.905	0.009	0.264	0.792	−3.233	4.240
Gastrointestinal	1.312	2.058	0.019	0.638	0.524	−2.724	5.348
Source of disease information (reference category: healthcare provider)							
Family and friends	−7.232	1.565	−0.101	−4.620	**< 0.001**	−10.303	−4.162
Social media	−11.605	1.442	−0.174	−8.048	**< 0.001**	−14.433	−8.776
Country (reference category: Egypt)							
Jordan	10.300	1.076	0.253	9.571	**< 0.001**	8.189	12.411
Oman	3.081	1.325	0.062	2.325	**0.020**	0.482	5.681
Self‐care confidence	0.443	0.022	0.482	19.976	**< 0.001**	0.399	0.486

*Note:* *R* ^2^ = 0.36, adjusted *R* ^2^ = 0.35. Bold values are significant at *p* < 0.05.

Gender was a significant factor, with males demonstrating higher self‐care management scores than females (*B* = 0.103, *p* < 0.001). Age also showed a significant positive association with self‐care management, indicating that older patients tended to engage more effectively in self‐care management (*B* = 4.349, *p* < 0.001). Regular healthcare visits were associated with lower self‐care management scores (*B* = −1.805, *p* = 0.034), suggesting that those who frequently visited healthcare providers may rely more on professional care than on self‐management strategies. Among the disease categories, patients with diabetes mellitus exhibited significantly lower self‐care management scores than those with haematological conditions (*B* = −4.851, *p* = 0.005). Conversely, patients with renal disease reported significantly higher self‐care management scores (*B* = 6.387, *p* = 0.006). No significant differences were observed among patients with cancer, cardiovascular disease, cerebrovascular accidents, respiratory diseases, or gastrointestinal disorders. Health information is a significant determinant of self‐care. Patients who relied on family and friends (*B* = −7.232, *p* < 0.001) or social media (*B* = −11.605, *p* < 0.001) demonstrated significantly lower self‐care management scores than those who received information from healthcare providers. Geographic differences were also notable, with patients from Jordan (*B* = 10.300, *p* < 0.001) and Oman (*B* = 3.081, *p* = 0.020) exhibiting higher self‐care management scores than those from Egypt. Among all predictors, self‐efficacy emerged as the strongest determinant of self‐care management (*B* = 0.443, *p* < 0.001), reinforcing the crucial role of self‐efficacy in effective disease management.

## 5. Discussion

This study assessed self‐care behaviours among patients with chronic diseases using the Self‐Care of Chronic Illness Inventory. The results revealed that patients reported relatively low mean scores across all self‐care behaviour subscales: self‐care maintenance (*M* = 60.8, SD = 16.9), self‐care monitoring (*M* = 64.6, SD = 19.8) and self‐care management (*M* = 66.8, SD = 19.9). These findings align with those of previous studies that have documented suboptimal self‐care behaviours in patients with chronic illnesses, particularly heart failure, diabetes and COPD [24–27].

Self‐care maintenance, which involves adherence to health‐promoting activities such as medication adherence, diet and physical activity, was the lowest among the subscales. This is concerning given that adequate self‐care maintenance has been shown to significantly reduce hospitalisation and improve long‐term outcomes in patients with chronic diseases [28]. Barriers such as financial constraints, a lack of motivation, and limited health literacy may explain these low levels [15].

Self‐care monitoring, which entails recognising symptoms and tracking disease progression, was slightly better than maintenance but still below optimal levels. These findings are consistent with previous research showing that patients often struggle to recognise the early warning signs of disease exacerbation [24, 29, 30]. This may be attributed to cognitive impairment, low educational levels, and a lack of structured monitoring programmes. Kessing et al. [25] highlighted that fatigue significantly impacts self‐care monitoring in heart failure patients, as exhaustion reduces the ability to detect early warning signs and act accordingly. Self‐care management, which refers to patients′ responses to symptom exacerbations, yielded the highest mean score among the three subscales but remained below the clinical threshold for optimal self‐care. This suggests that while patients may recognise symptoms, they often fail to take appropriate action because of uncertainty, lack of confidence, or over‐reliance on healthcare providers [3, 15]. A meta‐synthesis found that patients’ ability to manage symptoms is significantly influenced by their emotional state, with anxiety and depression acting as barriers to effective self‐care [26]. These findings underscore the urgent need for targeted interventions to enhance self‐care behaviour in patients with chronic illnesses. Healthcare providers should implement patient‐centred education programmes that address barriers, such as health literacy, motivation and emotional distress. Additionally, integrating digital self‐monitoring tools and strengthening social support systems may empower patients to play a more active role in managing their conditions, ultimately improving their long‐term health outcomes.

### 5.1. Predictors of Self‐Care Behaviours

Several key predictors of self‐care behaviour were identified in this study. Self‐efficacy emerged as the strongest determinant across all subscales, with higher levels being significantly associated with better self‐care maintenance, monitoring and management. This is consistent with the Middle‐Range Theory of Self‐Care, which posits that self‐efficacy is a critical factor in self‐care engagement [9, 29]. Similarly, Wu et al. [8] demonstrated that self‐efficacy fully mediates the relationship between knowledge and self‐care among patients with CKD, highlighting the need for interventions that enhance patients’ confidence in managing their health.

These findings can also be understood within a broader psychosocial and relational framework of self‐care. Emerging evidence suggests that self‐care behaviours are not solely determined by individual knowledge or skills but are strongly influenced by emotional distress, interpersonal dynamics and cognitive processes such as self‐efficacy. Psychological factors such as anxiety and depression have been shown to negatively affect engagement in self‐care behaviours, reducing motivation and adherence to treatment recommendations [31]. In addition, relational dynamics, including the quality of interactions with healthcare providers and the level of social support, play a critical role in shaping patients’ ability to effectively manage their condition [32]. Cognitive factors, particularly self‐efficacy, remain central in this process, as they mediate the relationship between knowledge, emotional state and behavioural engagement [33]. These findings align with recent evidence highlighting the multidimensional nature of self‐care, where behavioural outcomes emerge from the interaction between psychological, relational and contextual factors [31–33].

Educational attainment was another significant predictor, with individuals with a diploma or higher demonstrating superior self‐care maintenance and monitoring. These findings are consistent with previous research showing that higher education is associated with better health literacy, improved disease knowledge and greater adherence to self‐care behaviours [15, 24, 28]. By contrast, individuals with lower educational levels may struggle with disease comprehension and symptom recognition, contributing to poorer self‐care [28]. Furthermore, sex differences were observed in self‐care behaviours. Female participants had higher self‐care maintenance and monitoring scores than male participants, which aligns with previous studies reporting that women tend to be more proactive in managing their health [13, 34]. However, men demonstrate better self‐care management scores, suggesting that they may be more confident in responding to symptom exacerbations [29].

Age was a significant predictor of self‐care management, with older patients demonstrating better engagement in symptom management than younger ones. This finding aligns with previous research, indicating that older adults tend to develop structured routines, greater disease awareness, and more experience in recognising and responding to symptoms over time [35]. However, age was not a significant factor for self‐care maintenance and monitoring. One possible explanation is that while older adults may effectively manage acute symptoms, they might not consistently engage in proactive behaviours such as routine monitoring and preventive measures. Cognitive decline and functional impairments, which are more prevalent in ageing populations, can limit their ability to adhere to complex self‐care regimens, making them more dependent on carers or healthcare professionals for routine disease monitoring [9]. Additionally, studies have shown that older adults with multiple chronic conditions often experience greater difficulty balancing the self‐care requirements of different diseases, which can lead to the prioritisation of symptom management over maintenance activities [3]. Conversely, younger individuals may struggle with self‐care because of competing responsibilities, lower perceived vulnerability to disease complications, and a lack of established health routines [27]. The mixed influence of age on different aspects of self‐care highlights the need for age‐specific interventions that address not only acute symptom management but also long‐term self‐care behaviours, ensuring that both younger and older patients receive tailored education and support based on their unique challenges and capabilities.

Regular healthcare visits are associated with improved self‐care maintenance and monitoring, reinforcing the importance of continuous patient–provider interactions [15]. However, an unexpected negative association between regular healthcare visits and self‐care management suggests that patients who frequently visit healthcare providers may rely more on clinical interventions than actively engage in independent self‐management [3]. In addition, this may be because they may have experienced acute symptoms, which have been found to correlate with lower engagement in self‐care management [25].

Disease‐specific factors also play a significant role in predicting self‐care behaviours. Patients with cerebrovascular disease, cardiovascular disease, and diabetes exhibited significantly lower self‐care maintenance and monitoring scores than those with haematological conditions. This aligns with prior research highlighting that patients with multiple chronic conditions face complex self‐care challenges, often requiring assistance from carers or healthcare professionals [3, 30]. Conversely, patients with renal disease had significantly higher self‐care management scores, likely due to the structured nature of dialysis treatment, which reinforces self‐care behaviours [30]. In addition, the source of disease‐related information significantly affected self‐care behaviours. Patients who relied on healthcare providers as their primary source of information exhibited significantly better self‐care maintenance, monitoring, and management than those who obtained information from social media or family and friends. This finding suggests that professional guidance plays a crucial role in ensuring adherence to evidence‐based self‐care practices. In contrast, misinformation and inconsistent advice from non‐expert sources may contribute to poor self‐care behaviours, potentially leading to adverse health outcomes. These findings underscore the necessity of reinforcing credible provider‐led patient education and developing strategies to counteract misinformation, particularly in the digital age, where unverified health‐related content is widely accessible [14, 15]. Finally, the country of residence was a significant predictor of self‐care behaviours, with patients in Jordan and Oman reporting higher self‐care maintenance, monitoring, and management than those in Egypt. These variations may reflect differences in healthcare system structures, cultural attitudes towards chronic disease management, and economic conditions; however, these factors were not directly measured in the present study. Therefore, these interpretations should be considered potential explanations rather than definitive conclusions. Future research is needed to explicitly examine the role of healthcare system characteristics, socioeconomic factors, and cultural influences in shaping self‐care behaviours across countries. One contributing factor could be differences in national income levels, as Egypt is classified as a low‐income country, Jordan as a lower‐middle‐income country, and Oman as a high‐income country. Economic status influences healthcare accessibility, availability of self‐care resources, and the extent of patient education initiatives, all of which can impact self‐care engagement [15]. Additionally, healthcare policies and public health initiatives in each country may shape self‐care behaviours by determining the extent to which patients receive guidance regarding disease management and symptom monitoring. Cultural factors may also play a role, as some populations rely more on family support or healthcare providers than independently engaging in self‐care activities [9]. These findings highlight the need for strategies that address both healthcare access and educational disparities to promote self‐care in different economic and healthcare settings.

### 5.2. Limitations

Despite its strengths, this study has some limitations. This cross‐sectional design prevents the establishment of causality between predictors and self‐care behaviours. Future longitudinal studies should examine how self‐care behaviours evolve over time. Additionally, the use of self‐reported data may introduce recall and social desirability bias. Finally, psychosocial factors, such as depression, anxiety and carer burden, were not directly assessed despite their known impact on self‐care behaviours. Future studies should include these variables in more comprehensive analyses.

## 6. Implications for Practice

The findings of this study have important clinical and policymaking implications. Healthcare providers should focus on enhancing self‐efficacy, as confidence in self‐care abilities is the strongest predictor of better self‐care behaviour. Implementing motivational interviewing, structured self‐management programmes, and peer support groups can help to build patients’ confidence and competence in self‐care. Given the influence of education on self‐care behaviours, targeted health literacy interventions should be developed using simplified language, visual aids, and culturally tailored materials to improve understanding. Regular healthcare visits were associated with better self‐care monitoring and maintenance, suggesting that integrating self‐care education with routine medical consultation could enhance patient engagement. Furthermore, public health initiatives should focus on countering misinformation on social media by promoting credible health‐education platforms.

## 7. Conclusion

This study highlights suboptimal self‐care behaviours among patients with chronic diseases and identifies the key predictors of self‐care engagement. These findings emphasise the need for patient‐centred interventions that enhance self‐efficacy, improve health literacy and promote proactive self‐care behaviours. Future research should explore innovative self‐care strategies, such as digital health tools and behavioural coaching, to optimise patient outcomes.

## Author Contributions

Hanan Abdelrahman, Mohammad Al Qadire, Suha Ballout and Laura L. Hayman contributed to the study’s conceptualisation and methodology. Data collection was conducted by Hanan Abdelrahman, Hamada Zehry, Hala Aljezawi and Mohammad Al Qadire. Formal analysis and interpretation were carried out by Hanan Abdelrahman, Mohammad Al Qadire, Suha Ballout and Laura L. Hayman. The original draft of the manuscript was prepared by Hanan Abdelrahman and Suha Ballout and critical revision and editing were performed by Mohammad Al Qadire, Laura L. Hayman, Suha Ballout and Hamada Zehry.

## Funding

No funding was received for this manuscript.

## Conflicts of Interest

The authors declare no conflicts of interest.

## Data Availability

The data that support the findings of this study are available on request from the corresponding author. The data are not publicly available due to privacy or ethical restrictions.
